# In Silico and In Vitro Studies to Explore the Effect of Thymoquinone on Isocitrate Lyase, Biofilm Formation, and the Expression of Some Virulence Genes in *Candida albicans*

**DOI:** 10.3390/cimb46110771

**Published:** 2024-11-14

**Authors:** Masood Alam Khan, Mohd Azam, Hina Younus

**Affiliations:** 1 Department of Basic Health Sciences, College of Applied Medical Sciences, Qassim University, Buraidah 51412, Saudi Arabia; 2 Department of Medical Laboratories, College of Applied Medical Sciences, Qassim University, Buraidah 51412, Saudi Arabia; m.aftab@qu.edu.sa; 3 Interdisciplinary Biotechnology Unit, Faculty of Life Sciences, Aligarh Muslim University, Aligarh 202002, India; hinayounus@rediffmail.com

**Keywords:** black cumin, thymoquinone, virulence genes, infection, *Candida albicans*, molecular dynamics, biofilm, hyphae

## Abstract

Thymoquinone (TQ), a bioactive compound from black cumin (*Nigella sativa*), has demonstrated a broad range of therapeutic effects. The aim of this study is to evaluate the antifungal efficacy of TQ by targeting key virulence factors in *Candida albicans*, specifically focusing on isocitrate lyase (ICL) activity, biofilm formation, and gene expression. This study explored TQ’s impact on ICL, a decisive enzyme in the glyoxylate cycle, along with its effect on hyphal formation, biofilm development, and the virulent gene expression of *C. albicans* through in silico and in vitro studies. Molecular docking revealed a binding energy of −6.4 kcal/mol between TQ and ICL, indicating moderate affinity. The stability of the ICL-TQ complex was validated through 50 ns molecular dynamics simulations, showing the root mean square deviation (RMSD) values of 0.35 nm for ICL and 0.38 nm for the complex. In vitro studies further validated these findings, showing a dose-dependent inhibition of ICL activity. TQ at 2 µg/mL reduced enzyme activity by 57%, and at 4 µg/mL, by 91.4%. Additionally, TQ disrupted the yeast-to-hyphae switch, a key virulence factor, with 1 and 2 µg/mL doses significantly inhibiting hyphal formation. The biofilm formation was similarly affected, with a 58% reduction at 2 µg/mL and an 83% reduction at 4 µg/mL. TQ also downregulated the ALS1 and HWP1 genes that are associated with adhesion and biofilm development, demonstrating its broad-spectrum antifungal activity. These findings suggest that TQ is a promising candidate for antifungal therapies, targeting multiple virulence factors in *C. albicans* and potentially overcoming biofilm-associated drug resistance. Future research should focus on in vivo validation, optimization for clinical applications, and expanding its spectrum against other drug-resistant fungal species.

## 1. Introduction

The glyoxylate cycle, a variation in the TCA cycle, enables organisms to convert acetyl-CoA into anaplerotic compounds using isocitrate lyase (ICL), which bypasses decarboxylation to produce glyoxylate and succinate. This process is mainly found in plants and some microorganisms that include intracellular bacteria and fungi [1]. The glyoxylate cycle helps in the intracellular survival and pathogenesis of important pathogens, for instance *Mycobacterium tuberculosis*, *Salmonella typhimurium*, *Pseudomonas aeruginosa*, *Yersinia pestis*, *Candida albicans*, *Paracoccidioides brasiliensis*, and *Penicillium marneffei* [1,2].

Since ICL is not found in mammals, it is proposed to be a safe drug target to inhibit the intracellular proliferation of bacterial and fungal infections [2]. Several compounds, including heptapeptides, salicylanilide derivatives, thio benzanilide, pyruvate-isoniazid, 3-nitropropionamides, and isatinyl thiosemicarbazones, have been found to target the ICL enzyme in *M. tuberculosis*, while in *C. albicans*, brominated resorcinol, bromophenols, and hydroquinone derivatives have shown inhibitory effects on the same enzyme [3,4,5,6,7,8,9,10,11]. Itaconate has been shown to inhibit ICLs from *M. tuberculosis* and *Pseudomonas indigofera* [12,13]. Along with synthetic inhibitors, natural compounds like meroditerpenoids from brown algae, dihydroxystyrene metabolites, and hyrtiosin B from marine sponges have also demonstrated inhibitory effects on ICL activity in *C. albicans* [14,15,16]. The extracts of *Zingiber officinale* and *Illicium verum* have also been illustrated to inhibit ICL activity [17]. *C. albicans* uses a range of virulence factors that assist in the adherence and colonization of the fungal pathogen in host cells [18]. Of the various virulence factors, biofilm and hyphae formation are particularly potent contributors to the pathogenicity of *C. albicans*. Fungal adhesion, filamentation, and biofilm formation are controlled by genes such as the *Als* family and *Hwp1*, which play a vital role in biofilm development [19,20].

*Nigella sativa* (black seed) exhibits a wide range of therapeutic effects, including anti-inflammatory, antioxidant, antimicrobial, and anticancer properties [21]. *N. sativa* has been shown to support the immune system; reduce inflammation; combat infections caused by bacteria, fungi, and viruses; and inhibit cancer cell proliferation by inducing apoptosis and suppressing angiogenesis, making it a promising natural remedy for various conditions like diabetes, hypertension, and cancer [21]. Thymoquinone (TQ), one of the most abundant natural compounds in black seed, possesses potent antimicrobial activity against bacteria, fungi, and viruses [22]. Ahmad et al. demonstrated that TQ prevented the growth of *Escherichia coli* by impeding ATP synthase activity [23]. Moreover, TQ has been shown to inhibit the activity of P450 enzymes and mitogen-activated protein kinases (MAPKs) [21]. It inhibited SARS-CoV-2 protease activity, potentially disrupting the virus’s replication and propagation in host cells [24]. An in silico study by Tabbasum and Ahmad demonstrated that TQ can inhibit the functioning of Cag A and Vac A proteins of *Helicobacter pylori* [25].

This study is justified by the urgent need to explore novel antifungal agents like thymoquinone, which target multiple virulence factors in *C. albicans*, addressing the growing problem of drug resistance and biofilm-associated infections that are difficult to treat with conventional therapies. Here, in silico and in vitro effects of TQ were investigated on the activity of ICL and its consequence on *C. albicans* proliferation. As per the results, TQ inhibited ICL activity, halted hyphal development and intracellular growth, reduced biofilm formation, and suppressed *ALS1* and *HWP1* expression in *C. albicans* (graphical abstract). Given that ICL is absent in human cells, it presents a promising therapeutic target with minimal risk of host toxicity. Furthermore, TQ’s impact on biofilm formation addresses a critical challenge in *C. albicans* treatment, potentially enhancing the effectiveness of antifungal therapies. This research contributes to the growing need for alternative antifungal strategies, especially in the context of increasing drug resistance. The dual action of TQ in disrupting both metabolic pathways and structural defenses of *C. albicans* underscores its potential as a novel therapeutic agent in combating invasive fungal infections.

## 2. Materials and Methods

### 2.1. Docking Analysis of TQ and Its Interaction with ICL Protein Target

ICL and TQ were analyzed using AutoDock Vina to predict their binding mode, identifying key interactions and the optimal conformation of the TQ-ICL complex [26]. The RCSB Protein Data Bank (PDB) provided ICL structure, identified by PDB ID: 1DQU. The docking process involved selecting a monomeric subunit of the ICL structure. The removal of pre-bound ligands and solvent molecules, alongside the inclusion of polar hydrogens and assignment of Kollman charges, was carried out using MGLTools-1.5.6. The molecular docking grid center was positioned at X = 48.451, Y = 28.475, and Z = 19.421, with grid box dimensions of 86 × 62 × 104 Å^3^ and 1 Å spacing to encompass all ICL active site residues. TQ’s structure (CID: 10281) was downloaded from the PubChem database in sdf format and underwent potential energy reduction to achieve optimized geometry. Following minimization, the TQ structure was saved in pdb format via Chimera-1.10.2 to facilitate its application in docking experiments [27].

The BFGS (Broyden–Fletcher–Goldfarb–Shanno) algorithm was employed to dock ICL and TQ, with the exhaustiveness parameter set to 100 to ensure a thorough search. To maintain standard conditions, all additional docking parameters were left at their default configurations. A 2 Å RMSD threshold was applied for the docking evaluation during the clustering analysis to group similar binding poses and evaluate the binding interactions more comprehensively. A detailed 2D schematic of the interacting residues in the ICL-TQ complex was created using the Discovery Studio Visualizer (Accelrys [2.1], 2008), offering a clear representation of the molecular interactions. Additionally, a 3D visualization and structural analysis of the residues in the ICL-TQ complex were conducted using Chimera-1.10.2 [25], allowing for a detailed examination of the ligand–enzyme spatial interactions.

### 2.2. Simulation Studies Using Molecular Dynamics (MD) to Examine the ICL-TQ Complex

MD simulation was executed on ICL and the ICL-TQ complex with the highest binding affinity using Gromacs-2018.1 and the Amber99SB-ILDN force field in an aqueous environment [28,29]. After isolating TQ from the ICL-TQ complex, Antechamber and the AM1-BCC charge model in AmberTools21 were used to generate its topology [30]. A triclinic box, filled with TIP3P water, was employed to solvate the ICL and ICL-TQ complex, followed by neutralization with Na^+^/Cl^−^ ions and energy minimization via the steepest descent algorithm for 5000 steps. The system was equilibrated in the NVT ensemble at 300 K for 200 ps, utilizing a V-rescale thermostat, followed by NPT equilibration at 1.0 bar and 300 K with a Parrinello–Rahman barostat, both for 200 ps [31,32]. A cutoff value of 1.4 nm was applied for Coulomb and Lennard–Jones interactions, with an integration time step of 2 fs [33]. The system’s electrostatic interactions were modeled with the PME method, applying a Fourier transformation grid spacing of 0.16 nm [34]. For both ICL and the ICL-TQ complex, production MD simulations were run for 50 ns, generating 5000 frames per trajectory. After PBC corrections, GROMACS utilities were used for the analysis, and MM-PBSA calculated binding energies for the ICL-TQ complex during the MD simulation [35].

### 2.3. Candida Albicans

The *Candida albicans* strain from the American Type Culture Collection (ATCC 60193) was generously supplied by the Microbiology Laboratory of the College of Applied Medical Sciences at Qassim University, Buraydah, Saudi Arabia. Upon receipt, the strain was carefully cultured on Sabouraud Dextrose Agar (SDA from HiMedia, Mumbai, India) to ensure optimal fungal growth and viability. SDA, a widely used medium for cultivating fungi, provided the necessary nutrients for *C. albicans* proliferation, allowing for subsequent experiments and analyses.

### 2.4. Effect of TQ on Isocitrate Lyase (ICL) Enzyme Activity of C. albicans

To enhance ICL expression, *C. albicans* was grown in YNB with 2% sodium acetate, followed by centrifugation and PBS washing. *C. albicans* cells were lysed in a lysis buffer followed by its sonication for 1 min [36]. Once centrifuged at 10,000× *g*, the supernatant was taken for the ICL activity analysis. The reaction included a 20 mM sodium phosphate buffer (pH 7.0), 1 mM threo-DL (+) isocitrate, 5 mM MgCl_2_, 4 mM phenylhydrazine, and *C. albicans* lysate. The reaction mixture was incubated at 37 °C, with various concentrations (1, 2, and 4 µg/mL) of TQ (Sigma-Aldrich, St. Louis, MO, USA) or without, and the activity of ICL was monitored at 324 nm.

### 2.5. Determination of Anti-C. albicans Efficiency of TQ

The effect of TQ on *C. albicans* was assessed through the agar well diffusion and dilution techniques [37]. To assess the antifungal activity of TQ via agar well diffusion, a *C. albicans* inoculum was seeded onto SDA plates, with 10 mm diameter wells created, each loaded with TQ (50, 100, and 200 µg). Followed by the incubation for 24 h, the inhibition zone was used as a metric to assess TQ’s anti-*Candida* activity.

As outlined by the National Committee for Clinical Laboratory Standards (NCCLS) guidelines, the MIC of TQ for *C. albicans* was measured using the macrodilution method. Serial dilutions of TQ were prepared, inoculated with *C. albicans*, and incubated, with the MIC noted as the lowest concentration that visibly inhibited *C. albicans* growth [38]. The antifungal susceptibility testing was performed in glass tubes containing 5 mL of Sabouraud dextrose broth (SDB, HiMedia, Mumbai, India). TQ (ranging from 0.50 to 128 μg/mL) and the *C. albicans* inoculum (1 × 10^5^ cells/100 µL) were added to each tube. The MIC value, indicating the lowest concentration that stopped visible fungal growth, was measured after incubating for 24 h. The minimum fungicidal concentration (MFC) of TQ was calculated by plating 50 µL of the suspension from all tubes onto SDA. After incubating for 24 h, the plates were examined for *C. albicans* growth, and the concentration of TQ that entirely suppressed growth was noted as the MFC.

### 2.6. Time-Kill Assay for Evaluating C. albicans Inhibition Efficiency by TQ

After culturing *C. albicans* in SDB for 24 h at 37 °C, TQ’s activity against the fungus was tested using the previously reported time-kill method [39]. Each flask, containing 20 mL of SDB and 0.5, 1, 2, and 4 µg/mL of TQ, was inoculated with *C. albicans* cells (5 × 10^5^), and 100 µL of the samples were taken at 0, 3, 6, 12, and 24 h, followed by a 20 min spin at 10,000× *g*. After reconstitution in sterile PBS to remove TQ carryover, CFUs were examined by plating serial dilutions on SDA.

The intracellular growth of *C. albicans* with TQ was examined in J774 macrophages (1 × 10^6^ cells/well) cultured in 24-well plates with DMEM (Sigma Aldrich, St. Louis, MO, USA) and 10% FBS. Following 24 h, macrophages were exposed to *C. albicans* in a 1:2 ratio. Unphagocytosed *C. albicans* was removed by rinsing with PBS after 4 h of incubation, followed by 24 h treatment of infected macrophages with 2 and 4 µg/mL TQ, washing, and lysis with 1% Triton X-100 to release intracellular *C. albicans.* Lysates from each sample were diluted, plated on SDA, and CFUs were counted, with final values calculated based on the dilution factor.

### 2.7. Inhibitory Effect of TQ on Hyphae Formation

The incubation of *C. albicans* with fetal bovine serum occurred with or without TQ (1 and 2 µg/mL) for 6 h [37]. A small amount of the cell suspension from each sample was placed on a slide for the microscopic observation of hyphae formation at ×100 magnification.

### 2.8. Inhibitory Effect of C. albicans Biofilm Formation by TQ

The inhibition of *C. albicans* biofilm formation by TQ was evaluated using a previously reported protocol [39]. A suspension of 1 × 10^6^ cells/mL of *C. albicans* in SDB was incubated in a 96-well plate with 1 × 10^5^ cells and 2 or 4 µg/mL TQ for 48 h. After a gentle PBS wash and drying, 0.1% crystal violet was applied to the wells for 10 min, then rinsed and allowed to dry. Finally, ethanol (100 µL) was included into each well to solubilize biofilm-bound crystal violet. A BIO-TEK EL310 Microplate Auto-Reader was used to measure absorbance at 595 nm.

### 2.9. Determination of Virulence Gene Expressions ALS1 and HWP1 by RT-PCR

In *Candida albicans*, *ALS1* and *HWP1* expression was assessed to determine their roles in biofilm development and adhesion [19]. The fresh culture of *C. albicans* was mixed with 1 mL fresh SDB, and different TQ concentrations (0.25 to 2 µg/mL) were added as the drug in four different tubes, and one tube without TQ was considered as a control. The samples, incubated for 24 h, were centrifuged at 5000 rpm for 10 min, and the pellet was re-suspended in 400 µL of a 1X TE buffer (pH 8.0), and vortexed.

According to the manufacturer’s protocol, a TRIzol™ reagent (Invitrogen, Thermo Fisher, Waltham, MA, USA) was employed for total RNA extraction, and the extracted mRNA was subsequently dissolved in 10 μL of nuclease-free water. The reverse transcription of 500 ng total RNA was accomplished with the Maxima First Strand cDNA Synthesis Kit (Invitrogen, Thermo Fisher), in compliance with the manufacturer’s recommendations. mRNA amplification by PCR was performed using Dreamtaq Green PCR Master Mix (Invitrogen, Thermo Fisher) with primers detailed in Table 1.

The PCR process involved an initial 4 min denaturation at 94 °C, 35 cycles of 94 °C for 30 s, 52 °C for 1 min, 72 °C for 1 min, and a final 7 min extension at 72 °C. PCR products were separated on a 2% agarose gel post-amplification, stained with ethidium bromide, and visualized under UV light to confirm the expected DNA fragment size and amplification specificity.

### 2.10. Statistical Analysis

The data were analyzed using one-way ANOVA followed by a Bonferroni post hoc test. A *p*-value of <0.05 was considered statistically significant. The values are displayed as the mean ± S. D. of three independent values.

## 3. Results and Discussion

### 3.1. Binding Interaction Analysis Between ICL and TQ Through Molecular Docking

**Figure 1:**
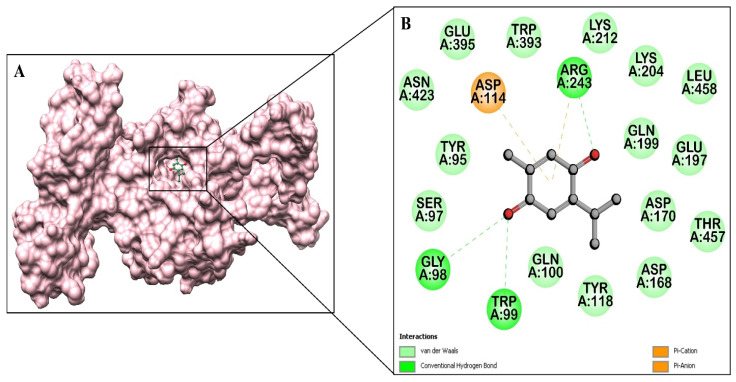
(**A**) Three-dimensional structure of isocitrate lyase (ICL) (PDB ID: 1DQU) in complex with TQ. (**B**) Two-dimensional schematic of ICL residues interacting with TQ.

The docking results from AutoDock Vina revealed a Gibbs free energy of −6.4 kcal/mol for the ICL-TQ interaction, signifying a stable binding affinity. This implies that TQ could potentially interfere with the function of ICL. The docking procedure generated 10 different binding poses, with the most stable and energetically favorable conformation (Figure 1A). With a calculated binding constant (Kb) of 4.91 × 10^4^ M^−1^, the ICL-TQ complex exhibits moderate binding strength between the interacting molecules. TQ interacts with the ICL protein through three hydrogen bonds, specifically with the Gly98, Trp99, and Arg243 residues (Figure 1B). TQ’s aromatic ring is engaged in π interactions with Arg243, and the residues Tyr95, Ser97, Gln100, Tyr118, Asp168, Asp170, Glu197, Gln199, Lys204, Lys212, Trp393, Glu395, Asn423, Thr457, and Leu458 have van der Waals interactions with TQ. In addition, the Asp114 residue of ICL is engaged in Π (pi)–anion with the aromatic ring of TQ. The residues Asp114, Asp168, Asp170, and Glu197 comprise the catalytic site for binding of the ICL substrate, suggesting that the binding of TQ to these residues will impart the inhibitory effect in the activity of this enzyme [40]. As a result, the interaction between TQ and ICL is favorable and well stabilized by hydrogen-bonding, hydrophobic, van der Waals, and π–π interactions.

### 3.2. MD Simulations

#### 3.2.1. Analysis of Residue Dynamics and Structural Deviations

**Figure 2:**
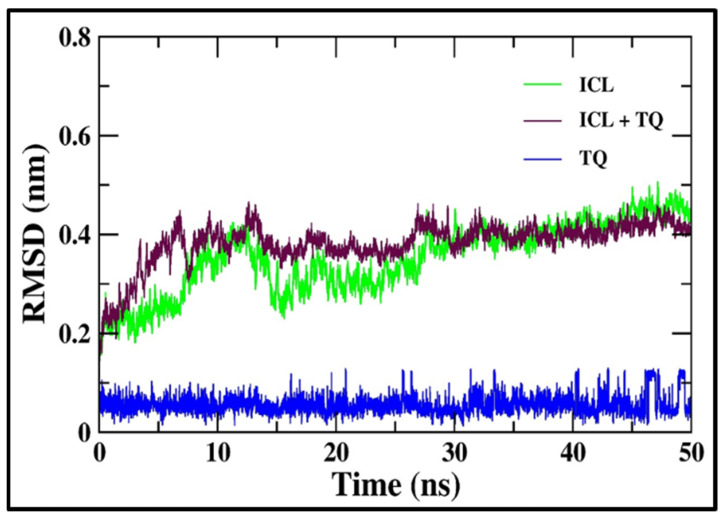
The root mean square deviation (RMSD) trajectory of ICL’s backbone atoms, both unbound and in complex with TQ, was analyzed over the 50 ns MD simulation to track structural changes.

Multiple GROMACS utilities were employed to examine the MD simulation trajectories of both ICL and the ICL-TQ complex. The average root mean square deviation (RMSD) of the backbone for both ICL alone and ICL-TQ was determined in comparison to their initial conformations (Figure 2). Following 25 ns of equilibration, the RMSD of ICL and the ICL-TQ backbone atoms stabilized and exhibited minimal fluctuations throughout the remaining MD simulation. RMSD calculations showed average values of 0.35 nm for ICL and 0.38 nm for the ICL-TQ complex, indicating that the structural deviations in both systems were minimal throughout the simulation. A gradual rise in RMSD for the ICL-TQ complex suggests that the binding of TQ introduced only minor conformational changes, maintaining overall stability of the protein–ligand complex. Being indicated in Figure 2, the binding of thymoquinone results in only minor conformational shifts in ICL. RMSD values, consistently under 0.5 nm, confirm that the binding process does not cause significant deviations from the initial conformation, and such values are generally considered appreciable in molecular dynamics studies [41,42,43]. RMSD values of ICL with TQ confer a stable nature of interaction for the ICL-TQ.

**Figure 3:**
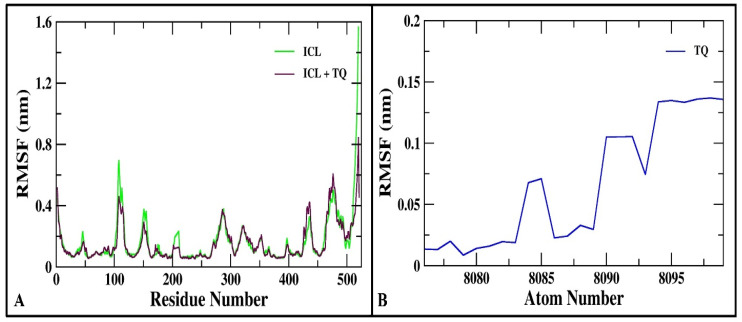
(**A**) RMSF plot of backbone atoms across residues for ICL alone and in complex with TQ. (**B**) RMSF of atoms of TQ.

Additionally, an RMSF analysis was conducted, as shown in Figure 3A. The majority of residues in both ICL and the ICL-TQ complex showed backbone RMSF values below 0.5 nm. The elevated fluctuations in residues 105–115 are credited for by the protein loop region, which is inherently more flexible. The interaction with TQ slightly reduces the fluctuation of loop residues in ICL. Therefore, the residue-specific RMSF plot of the ICL backbone atoms indicates that binding with thymoquinone does not significantly alter the fluctuation profile. The RMSF values for the atoms of thymoquinone are presented in Figure 3B.

#### 3.2.2. Evaluating the System’s Stability, Degree of Compactness, and Overall Energy Profile

**Figure 4:**
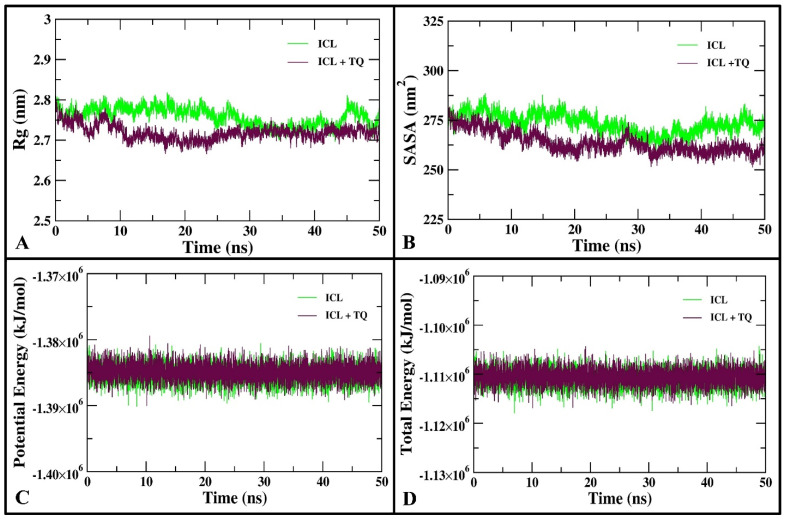
(**A**) The Rg analysis of the backbone atoms of ICL and the ICL-TQ complex over the course of the 50 ns MD simulation, reflecting their structural compactness and stability. (**B**) Time-resolved SASA of ICL and the ICL-TQ complex, demonstrating fluctuations in solvent exposure. (**C**) Potential energy and (**D**) total energy plots of ICL and ICL-TQ systems over the 50 ns MD simulation, illustrating the energy stability of both systems.

Protein stability in MD simulations is commonly evaluated by calculating the radius of gyration (Rg), which measures the RMS distance of atoms from the center of mass. By providing a mass-weighted value, this measurement reveals the protein’s compactness and structural behavior throughout the simulation. Smaller Rg values indicate a more compact structure, while changes in Rg may suggest the onset of unfolding or instability. Therefore, monitoring Rg is crucial for understanding how the protein’s conformation evolves during MD simulation and whether it maintains its native-like structure [44]. The Rg profile of the backbone atoms (Figure 4A) affirms that ICL retains structural stability and compactness during its interaction with TQ. The stable Rg values of both ICL and the ICL-TQ complex indicate minimal disruption to ICL’s structural dynamics during binding. With average Rg values of 2.75 nm for ICL alone and 2.72 nm for the ICL-TQ complex, the slight reduction in Rg after TQ binding suggests that the overall structural integrity of ICL is preserved. This small decrease reveals that the binding of TQ promotes a more compact protein conformation, without causing significant destabilization or unfolding. The reduction in Rg reflects an increased structural tightness, pointing to enhanced stability of the ICL-TQ complex compared to ICL alone. Over the 50 ns MD simulation, this suggests that TQ binding exerts a stabilizing effect on the protein’s structural dynamics, ensuring that ICL maintains its functional form and overall stability throughout the simulation period. These findings underline the role of TQ in reinforcing the compactness and structural resilience of ICL during molecular interactions [45].

Using the gmx sasa utility, an SASA analysis was conducted to examine the alterations in the hydrophobic core and solvent accessibility of ICL due to TQ binding. This evaluation provides insight into how TQ binding modulates the surface exposure of ICL, potentially altering the accessibility of hydrophobic regions to solvent molecules [46]. Throughout the entire 50 ns MD simulation, the SASA values for ICL and the ICL-TQ complex were calculated and shown in Figure 4B. The average SASA for ICL alone was 273.4 nm^2^, while for the ICL-TQ complex, it decreased to 263.7 nm^2^, suggesting reduced solvent accessibility due to TQ binding. The binding of TQ to ICL’s hydrophobic core led to minimal changes in solvent accessibility, with SASA values showing no significant deviations during the MD simulation. This indicates a stable interaction, preserving the structural integrity of ICL. The steady potential and total energy profiles of both the unbound ICL and the ICL-TQ complex throughout the simulation indicate a stable system, reinforcing the structural stability and integrity of the protein and complex (Figure 4C,D).

### 3.3. Molecular Mechanics Poisson–Boltzmann Surface Area (MMPBSA) Calculations Quantified the Energy Contributions Driving the ICL-TQ Complex Stability

Binding energy contributions for the ICL-TQ interaction were determined via MM-PBSA calculations, conducted on 250 frames sampled from the final 25 ns of the MD simulation. The protein–ligand complex was primarily stabilized through non-covalent interactions [47]. Electrostatic forces, van der Waals interactions, hydrophobic contacts, and hydrogen bonds play roles in ligand–protein binding, influencing stabilization or destabilization [42,48]. Table 2 lists the binding energies for the ICL-TQ complex, indicating that van der Waals interactions predominantly favor TQ binding, with SASA energy playing a secondary, moderate role. Electrostatic interactions made only a small contribution to the binding affinity between TQ and ICL, whereas polar solvation energy had a negative impact, counteracting the favorable interactions. This indicates that, although electrostatic forces are present, the high polar solvation energy creates an obstacle to effective binding. The continuum solvent’s interference with the ICL-TQ interaction leads to the negative contribution of polar solvation energy. Relative binding energy for this interaction was calculated to be −47.012 ± 1.22 kJ mol^−1^.

### 3.4. Thymoquinone Displayed Antifungal Efficacy Against C. albicans

**Figure 5:**
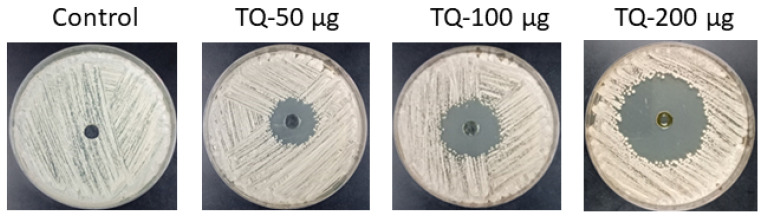
Assessment of TQ’s antifungal activity against *C. albicans* on SDA plates. Following incubation for 24 h at 37 °C, inhibition zones were measured in wells containing 50, 100, and 200 µg of TQ.

TQ exhibited potent antifungal activity against *Candida albicans*, as demonstrated by the zones of growth inhibition on SDA plates (Figure 5). At a concentration of 50 µg, TQ produced a clear inhibition zone measuring 21 mm, indicating significant fungal suppression. With the gradual increase in TQ concentration, the anti-Candida activity became more pronounced, with 100 µg of TQ creating a 29 mm inhibition zone and 200 µg leading to an even larger zone of 41 mm. These results suggest that the antifungal efficacy of TQ is dose-dependent, with greater inhibition observed at higher concentrations, underscoring its potential as an effective antifungal agent against *C. albicans*.

The agar well diffusion method revealed clear zones of inhibition, indicating the direct antifungal effect of TQ on *C. albicans*. Meanwhile, the dilution method allowed for a more precise quantification of TQ’s inhibitory and fungicidal properties, resulting in a minimum inhibitory concentration (MIC) of 2 µg/mL, the smallest concentration required to inhibit visible fungal growth. Additionally, the minimum fungicidal concentration (MFC) was determined to be 4 µg/mL, demonstrating the concentration at which TQ successfully killed the fungal cells. These findings highlight TQ’s potent antifungal properties against *C. albicans*.

### 3.5. TQ Demonstrated Significant Inhibition of C. albicans ICL Activity

**Figure 6:**
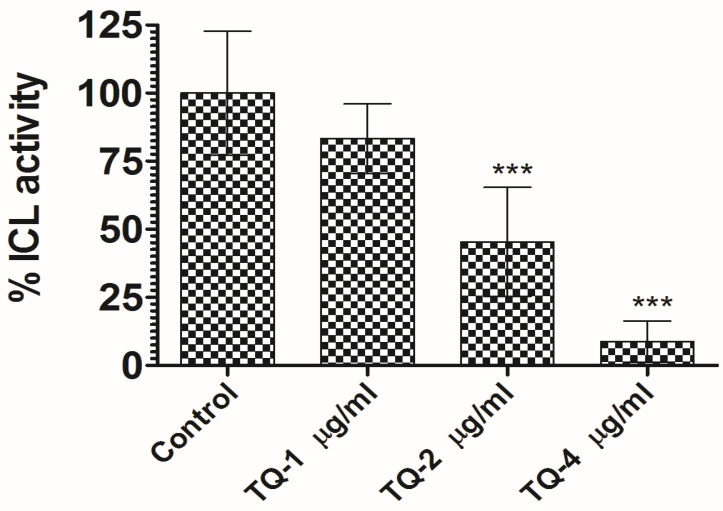
TQ inhibits the activity of ICL in *C. albicans*. ICL activity in *C. albicans* lysate was assessed by measuring absorbance at 324 nm after a 30 min incubation with TQ at 1, 2, and 4 µg/mL. A significant reduction in ICL activity was observed with increasing TQ concentrations, with *** (*p* < 0.001) indicating highly significant inhibition when compared to the control.

The inhibition of *C. albicans* ICL by TQ occurred in a dose-dependent fashion (Figure 6). At 2 µg/mL, enzyme activity decreased by 57%, and at 4 µg/mL, it showed a 91.4% decrease, in comparison to the untreated control (Figure 6) (*p* < 0.001). This evidence implies that TQ induces a marked loss of ICL activity, which is vital for pathogen virulence. In particular, ICL disruption hindered the persistence of *M. tuberculosis* during infection [49]. Moreover, ICL is also a key factor that contributes to *S. typhimurium* and *P. aeruginosa* pathogenicity [50,51]. *Aspergillus fumigatus*’ ability to utilize alternative carbon sources such as ethanol, acetate, and fatty acids, which is dependent on ICL, enables the pathogen to adapt to nutrient-limited environments, supporting its survival and pathogenicity [52]. Earlier studies have demonstrated that ICL is crucial for *C. albicans* virulence and blocking it can weaken the progression of candidiasis in mice [53]. Interestingly, previous studies have not explored the effect of TQ on ICL activity. Our findings demonstrate that TQ not only inhibits the enzymatic activity of *C. albicans* ICL, but also effectively halts the growth of *C. albicans*, highlighting its dual role in disrupting both metabolic function and fungal proliferation.

### 3.6. TQ Displayed Antifungal Effects in Both In Vitro Assays and Against Intracellular C. albicans

**Figure 7:**
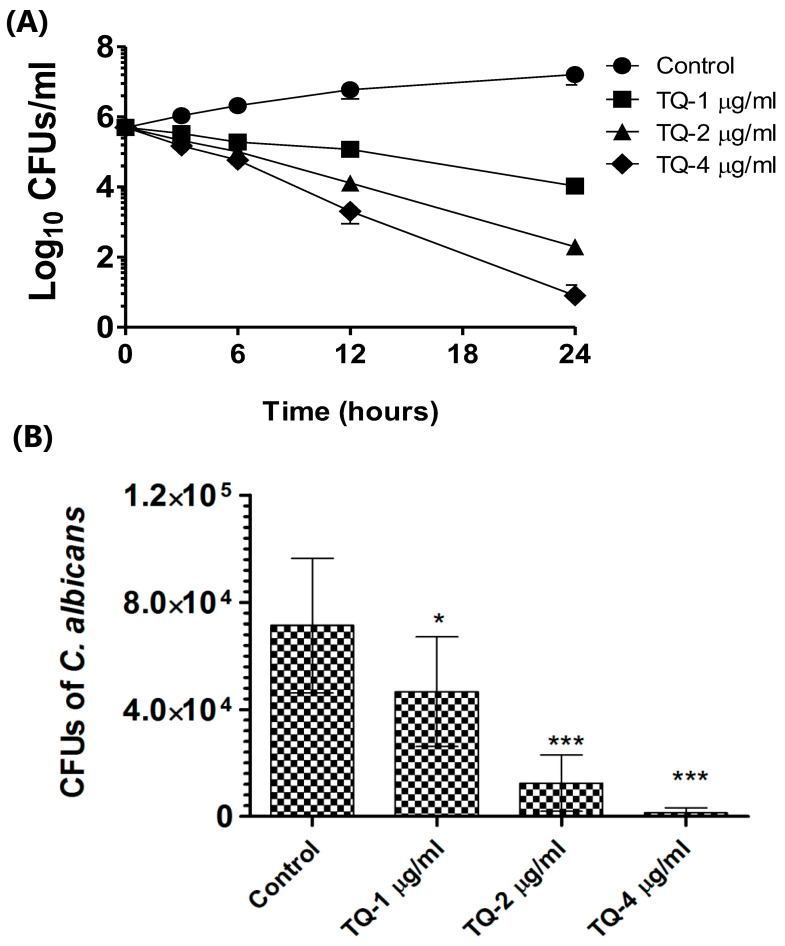
(A) TQ time-kill study against *C. albicans*. (B) TQ’s activity against *C. albicans* in J774 macrophages. Infected macrophages (1 × 10^6^ cells/well) were incubated with separate TQ concentrations for 24 h. Lysates underwent washing and lysis, were plated on SDA, and were incubated for 24 h, and CFUs were subsequently counted. Mean ± SD of three independent replicates was used to represent findings. * (*p* < 0.05), *** (*p* < 0.001).

TQ’s antifungal effectiveness against *C. albicans* was measured through time-kill studies, and CFUs were measured in TQ-treated and control groups to determine its killing effect. TQ blocked the multiplication of *C. albicans* in a time-dependent way (Figure 7A). When applied at 1 µg/mL, TQ killed about 97.9% of the starting inoculum, with higher doses of 2 and 4 µg/mL achieving 99.9% killing within 24 h (*p* < 0.001).

*Candida albicans* can multiply intracellularly as shown by its replication in macrophages. The load of *C. albicans* was found to be 71,302 ± 10,136 CFUs in the untreated macrophages (Figure 7B). TQ treatment decreased the numbers of the intracellular yeast cells in macrophages (Figure 7B). The *C. albicans* cell count dropped to 46,657 ± 8248 with 1 µg/mL TQ treatment, significantly lower than the 71,302 ± 10,136 in the control group (*p* < 0.05). TQ at 2 µg/mL reduced CFUs to 12,474 ± 4243, while 4 µg/mL resulted in a further drop to 1324 ± 774 (*p* < 0.001), demonstrating a substantial reduction. This showed that TQ treatment had higher efficacy against the intracellular replication of the pathogen. The engulfment of *C. albicans* by macrophages leads to cytokine secretion, which facilitates hyphae formation in the yeast cells [54]. In *C. albicans*, phagocytosis triggers an upregulation of ICL and malate synthase expression. Research highlights that ICL is key to *Candida glabrata*’s ability to survive inside macrophages by promoting metabolic adaptation, allowing the pathogen to thrive in nutrient-limited conditions and evade immune responses [55]. ICL also plays a role in ergosterol and chitin synthesis as *C. albicans* mutants lacking ICL had lower levels of these components [56]. This evidence supports the application of ICL inhibitors as a promising tool for combating drug resistance in *C. albicans*. In addition, an ICL mutant of *C. albicans* showed a lower expression of drug resistance genes such as ERG11 and CDR2 [57]. It suggests that the inhibition of ICL can aid in fighting the drug resistance in *C. albicans*.

### 3.7. TQ Suppressed Hyphal Formation in C. albicans, Thereby Decreasing Its Invasive Potential

**Figure 8:**
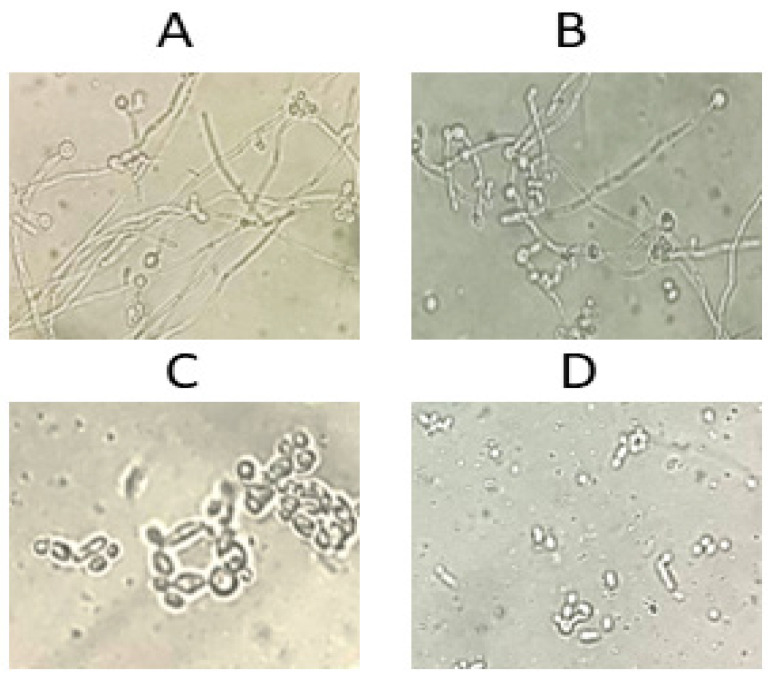
Effect of varying TQ doses on yeast-to-hyphae conversion in *C. albicans*: (**A**) control, (**B**) 1 µg/mL, (**C**) 2 µg/mL, (**D**) 4 µg/mL. Scale bar is 20 µm for all images.

Thymoquinone (TQ) demonstrated a potent ability to inhibit the yeast-to-hyphae transition in *Candida albicans*, a process crucial for its virulence and immune evasion. In untreated controls, *C. albicans* exhibited robust hyphal formation, which contributes to its pathogenicity (Figure 8A). At a concentration of 1 µg/mL, TQ partially inhibited hyphal formation, visibly reducing filamentous growth (Figure 8B). Increasing the concentration to 2 µg/mL further suppressed hyphal development, and at 4 µg/mL, TQ completely blocked hyphal formation, leading to cell death, demonstrating a clear dose-dependent inhibitory effect (Figure 8C,D). *C. albicans* can exist in both yeast and filamentous hyphal forms, with the latter being more virulent due to their ability to evade immune recognition. Hyphae are less detectable by immune cells like dendritic cells, which respond differently to yeast and hyphal forms, influencing cytokine production. The yeast form triggers IL-12 secretion, while hyphae stimulate IL-4 production, which weakens immune responses [58]. Unlike blastoconidia, *Candida albicans* hyphae are not recognized by Toll-like receptor 4 (TLR4) on peripheral blood mononuclear cells (PBMCs), preventing the activation of a typical immune response [59]. This allows the hyphal form to evade immune detection, contributing to the persistence and virulence of the infection. Additionally, *C. albicans* hyphae inhibit IFN-γ production by natural killer cells, further aiding in immune evasion [60]. By preventing the yeast-to-hyphae transition, TQ disrupts this immune evasion strategy, enhancing the host’s ability to recognize and eliminate *C. albicans* infections, positioning TQ as a promising antifungal agent.

### 3.8. TQ Impeded the Proficiency of C. albicans to Form Biofilm

**Figure 9:**
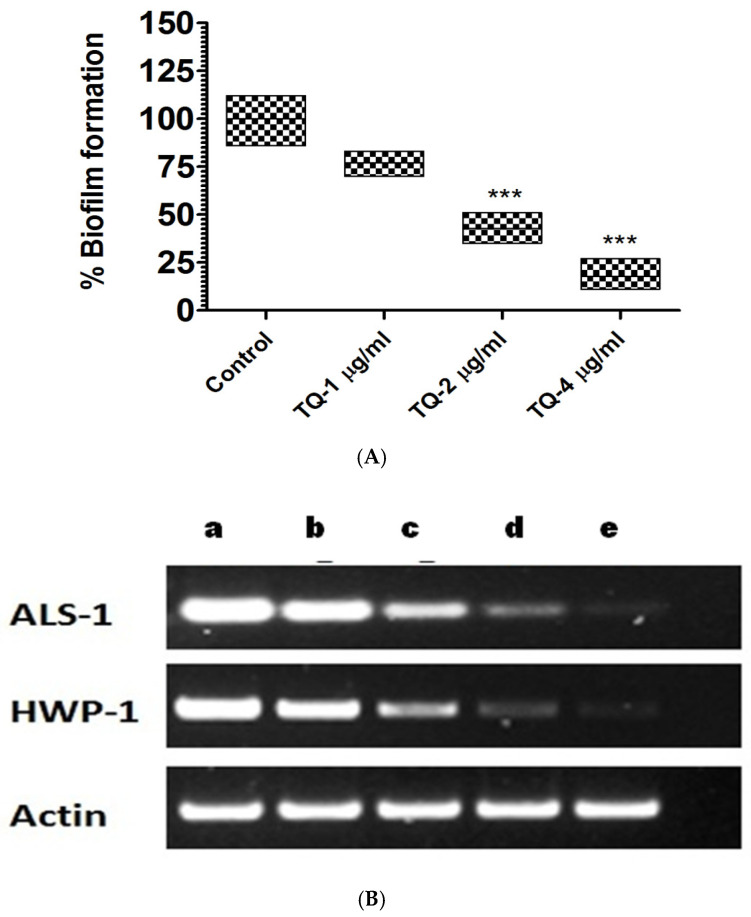
TQ exerts dose-dependent effects on biofilm formation and adherence gene expression. (**A**) TQ inhibits the formation of biofilm. *** (*p* < 0.001) TQ treatment vs. control. (**B**) TQ inhibits the expression of *ALS-1* and *HWP-1* molecules in *C. albicans*. (a) Control and (b) 0.25, (c) 0.5, (d) 1, and (e) 2 µg/mL. Equal loading was verified using actin as the control.

Biofilm formation serves as a protective measure for *C. albicans* against immune defenses and antifungal treatments, while simultaneously promoting its virulence and pathogenesis. At 2 and 4 µg/mL, TQ effectively reduced biofilm formation, reducing it by 58% and 83%, respectively, in comparison to the 100% biofilm formation in the untreated control (Figure 9A) (*p* < 0.001). *C. albicans* biofilms exhibited strong resistance to commonly used antifungal medications. The high tolerance is attributed to the presence of extrapolymeric substances and morphogenetic transition in biofilms. Moreover, the functional hyphae are required in the biofilm development that helps *C. albicans* to disseminate in host tissues.

Several key adhesion molecules, such as the Als protein family and HWP1, have essential roles in biofilm formation [61,62,63,64]. These molecules support *C. albicans* cells in adhering to surfaces and binding with each other, promoting the development of the dense biofilm matrix. HWP1, a glycosylphosphatidylinositol (GPI)-anchored mannoprotein, exhibits structural similarities to the Als proteins, both of which are essential for biofilm stability and integrity [65]. HWP1, in particular, acts as a substrate for transglutaminase enzymes, allowing the fungal pathogen to firmly adhere to host tissues, further enhancing its ability to form resilient biofilms not susceptible to antifungal therapy. The synergy between Als proteins and HWP1 underscores their pivotal role in mediating the adhesive properties and persistence of *C. albicans* biofilms. The data from this study demonstrated that TQ downregulated both *ALS1* and *HWP1* expression, with inhibition increasing in a dose-dependent manner (Figure 9B). TQ at 1 and 2 µg/mL led to a pronounced reduction in *ALS1* and *HWP1* expression. Earlier studies have demonstrated that, similar to *ALS1*, the *HWP1* gene also has a crucial role in biofilm formation as shown by an earlier study [66]. Key factors behind the drug resistance in *C. albicans* include the over-expression of drug-efflux pumps and the development of an extracellular matrix [67]. Drug-efflux pumps actively expel antifungal agents from the fungal cells, while the extracellular matrix works as a protective obstruction, reducing drug dispersion and increasing the biofilm’s overall resistance to treatment. Studies conducted earlier have revealed that TQ possesses the capability to inhibit biofilm formation in bacteria, fungi, and protozoans [68,69,70]. In the present study, TQ demonstrated significant efficacy in suppressing biofilm formation by *C. albicans*, implying that TQ influences not just cellular mechanisms but also disintegrates the complex biofilm structure that shields the fungal cells from antifungal agents. This finding highlights the ability of TQ to be an effective therapeutic agent to combat biofilm-related drug resistance in *C. albicans* infections.

## 4. Conclusions

In conclusion, this study provided compelling evidence that thymoquinone (TQ) exerts significant antifungal effects against *Candida albicans* by simultaneously targeting two vital virulence mechanisms: isocitrate lyase (ICL) activity and biofilm formation. ICL plays a crucial role in the glyoxylate cycle, enabling *C. albicans* to survive in nutrient-limited conditions, such as those encountered during host infection. By inhibiting this enzyme, TQ disrupts a key metabolic pathway essential for the fungus’s persistence and pathogenicity. Furthermore, biofilm formation in *C. albicans* enhances its resistance to antifungal agents and supports its ability to establish infections on various surfaces, including medical devices. TQ’s inhibitory action on biofilm formation thus represents an important breakthrough in limiting the protective shield that often renders *C. albicans* infections difficult to treat. The dual action of TQ on both metabolic (ICL inhibition) and structural (biofilm inhibition) aspects of *C. albicans* addresses the pathogen’s resistance mechanisms from multiple fronts, making it a highly promising therapeutic candidate. These findings align with the study’s broader aim of identifying novel antifungal strategies that go beyond the limitations of current treatments, which are increasingly compromised by drug resistance. The limitations of thymoquinone (TQ) for in vivo use include its poor bioavailability and rapid metabolism, which could limit its therapeutic efficacy when administered in living organisms. These limitations highlight the need for optimizing TQ formulations to enhance its stability and delivery for potential in vivo therapeutic use.

## Figures and Tables

**Table 1:** Details of primers for *ALS1*, *HWP1*, and actin of *C. albicans*.

Primer	5′-Sequence-3′	PCR Product Size (bp)	References
*ALS1*	Fwd: 5′-GAC TAG TGA ACC AAC AAA TAC CAG A-3′ Rev: 5′-CCA GAA GAA ACA GCA GGT GA-3′	318	[19]
*HWP1*	Fwd: 5′-ATG ACT CCA GCT GGT TC-3′ Rev: 5′-TAG ATC AAG AAT GCA GC-3′	572	[19]
Actin	Fwd: 5′-CCA GCT TTC TAC GTT TCC-3′ Rev: 5′-CTG TAA CCA CGT TCA GAC-3′	200	[20]

**Table 2:** Binding free energy (kJ mol^−1^) between thymoquinone (TQ) and Isocitrate lyase (ICL), as calculated by using Molecular mechanics Poisson–Boltzmann surface area (MM-PBSA).

	ICL-TQ Complex
ΔE_vdW_	−78.476 ± 1.097
ΔE_ele_	−5.548 ± 0.768
ΔE_PSE_	47.554 ± 1.587
ΔE_SASA_	−10.628 ± 0.109
ΔE_BE_	−47.012 ± 1.222

ΔE_vdW_: van der Waals interaction energy; ΔE_ele_: Energy from electrostatic interactions; ΔE_PSE_: Contribution from polar solvation energy; ΔE_SASA_: Energy due to solvent accessibility; ΔE_BE_: Overall binding energy.

## Data Availability

The original contributions presented in the study are included in the article, further inquiries can be directed to the corresponding author.
